# Novel Magnetic Countertraction Enhanced Performance in Colonic Endoscopic Submucosal Dissection: An Ex Vivo Crossover Study (With Video)

**DOI:** 10.1111/den.70120

**Published:** 2026-02-10

**Authors:** Hon Chi Yip, Wai Shing Chan, Siew Fung Hau, Nicole Miu‐yee Cheng, Louis Ho‐Shing Lau, Simon Chu, Zhaoyi Zhu, Man Yee Yung, Yee Kit Tse, Zheng Li, Simon Siu‐man Ng, Philip Wai‐yan Chiu

**Affiliations:** ^1^ Department of Surgery, Faculty of Medicine The Chinese University of Hong Kong Hong Kong SAR Hong Kong; ^2^ Faculty of Medicine, Institute of Digestive Disease The Chinese University of Hong Kong Hong Kong SAR Hong Kong; ^3^ Multi‐Scale Medical Robotics Center The Chinese University of Hong Kong Hong Kong SAR Hong Kong; ^4^ Department of Medicine and Therapeutics, Faculty of Medicine The Chinese University of Hong Kong Hong Kong SAR Hong Kong; ^5^ Li Ka Shing Institute of Health Sciences The Chinese University of Hong Kong Hong Kong SAR Hong Kong; ^6^ Medical Data Analytics Centre (MDAC) The Chinese University of Hong Kong Hong Kong SAR Hong Kong; ^7^ State Key Laboratory of Digestive Disease The Chinese University of Hong Kong Hong Kong SAR Hong Kong; ^8^ Chow Yuk ho Technology Center for Innovative Medicine The Chinese University of Hong Kong Hong Kong SAR Hong Kong

**Keywords:** colorectal neoplasia, endoscopic submucosal dissection, magnetic countertraction

## Abstract

Endoscopic submucosal dissection (ESD) is technically demanding with the main limitation on the lack of effective countertraction. Existing countertraction methods such as clip‐related techniques only provide unidirectional static traction force that may restrict their utility in complicated colorectal ESD. The magnetic countertraction system provides dynamic force by manipulating an external magnetic source. We designed a novel magnetic countertraction system with an internal magnet retractor introducible via the endoscopy channel and the external magnetic effector mounted on a robotic arm that could be easily manipulated. We evaluated the performance and safety of the system in an ex vivo randomized crossover study. ESD was performed on ex vivo porcine colon models with standardized 3 cm target lesions marked at gravity‐dependent locations. Endoscopists performed the ESD in pairs, randomized to magnetic countertraction (MAG‐ESD) or conventional ESD (C‐ESD) first to minimize bias from the learning effect. During MAG‐ESD, a flexible internal magnetic retractor was deployed via the endoscopic channel and anchored to the lesion margin. A robotic arm‐mounted external permanent magnet (EPM) was positioned above the colon model to engage the retractor and provide dynamic countertraction. Seventy‐two ESD (36 MAG‐ESD and 36 C‐ESD) were performed by 18 endoscopists. MAG‐ESD significantly reduced procedure time by 20.4% (*p* = 0.0002) and workload (NASA‐TLX mean difference: −19.81, 95% CI: −25.42 to −14.19). All procedures achieved en bloc resection. MAG‐ESD had significantly fewer complications (OR = 0.782, 95% CI: 0.644–0.949), including lower rates of perforation and muscle injury. The novel magnetic countertraction system significantly improved procedural efficiency, reduced operator workload, and enhanced safety in ex vivo colonic ESD.

## 1 Introduction

Endoscopic submucosal dissection (ESD) is the preferred resection technique for large superficial colonic neoplasia. In a large prospective cohort study, ESD resulted in a cure in 78.9% of the patients and a 5‐year intestinal preservation rate of 98.1% [1]. ESD is technically demanding without effective countertraction, leading to difficulty in exposing the dissection plane and a higher risk of adverse events. Various clip‐related traction techniques have been developed to provide tension for the dissection plane and optimal visibility during colorectal ESD [2, 3]. While these devices were shown to improve dissection efficiency, they could only provide static traction direction. In contrast, magnetic traction offers dynamic traction by externally manipulating an internal magnetic retractor. Previous research has explored various designs of magnetic retractors [4, 5, 6, 7]. The main drawback of most of these systems lay in the need for complete endoscope withdrawal and reinsertion, limiting the efficiency of the procedure. An alternative method described the use of an internal stainless steel anchor which could pass through the channel, but the retraction force generated is expected to be lower [8].

We have developed a novel magnetic countertraction system, consisting of an external permanent magnet (EPM) and the disposable internal magnetic retractor. The size of the internal retractor is compatible with 2.8 mm endoscopic channel, eliminating the need of scope withdrawal. The robot‐assisted EPM source also allows effortless and safe control of the external retractor. The system was tested in a live porcine pilot study, demonstrating the effectiveness of magnetic counteraction during gastric and rectal ESD [9]. In the current study, we aim to investigate the superiority of magnetic traction assisted versus conventional ESD through an ex vivo randomized crossover trial.

## 2 Procedure

### 2.1 Magnetic Countertraction System Design

**FIGURE 1:**
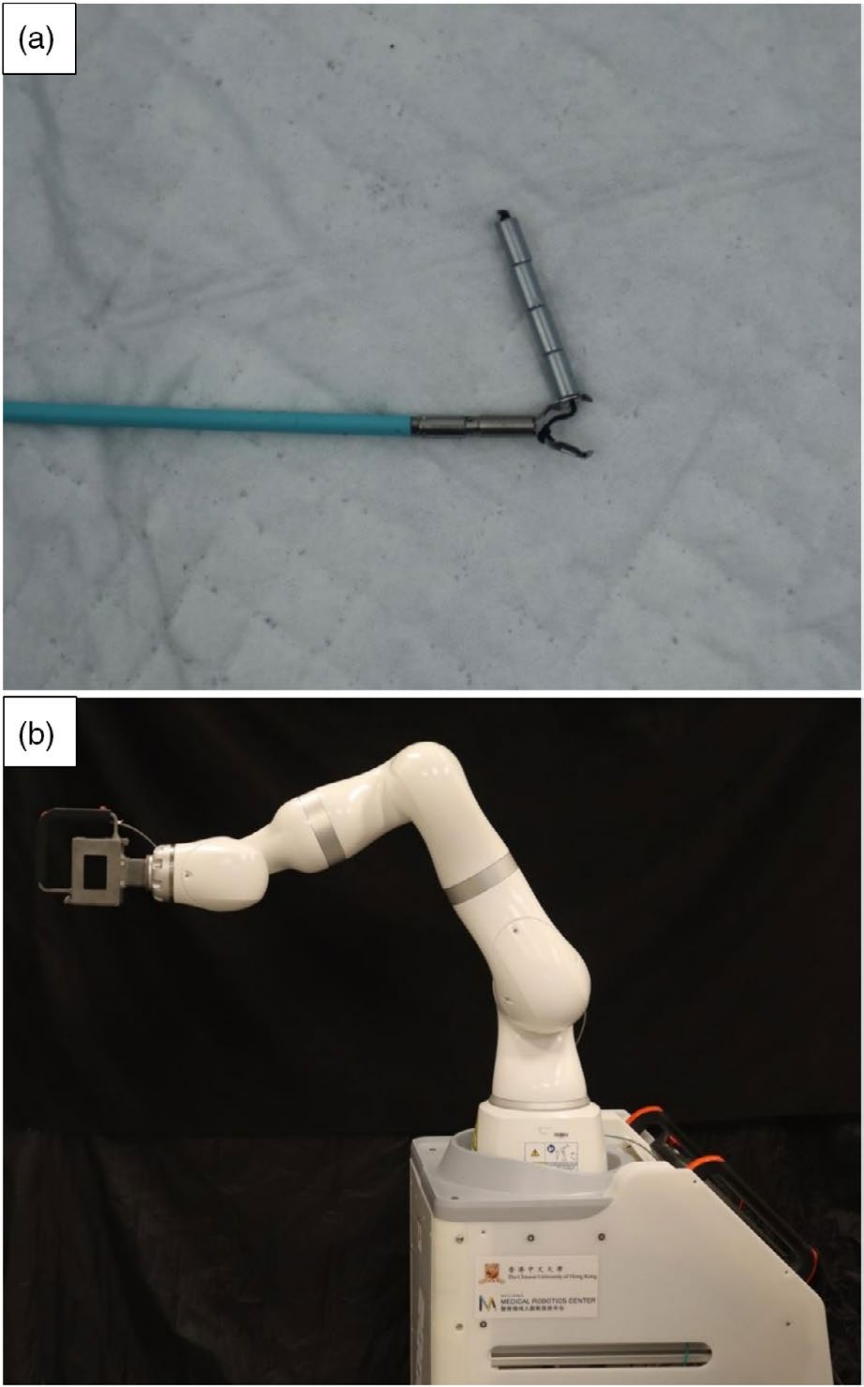
Magnetic countertraction system. (a) Internal magnetic retractor attached to endoscopic clip. (b) Robotic arm‐mounted external permanent magnet (EPM).

The internal magnetic retractor consisted of four small neodymium magnets with parylene coating (Each magnet with diameter 2.5 mm, length 5 mm, total magnetic flux density 273mT) and a repositionable endoclip (SD‐02‐230‐11C, Soudon Medical, China). The magnets were tied to one jaw of the clip by 3–0 silk braided suture (Figure 1). The design of the internal magnetic retractor is compatible with a 2.8 mm endoscopic channel and thus can be deployed without withdrawing the endoscope.

A robotic arm‐mounted EPM is used to provide a strong magnetic field for external traction force. The system composed of a permanent magnet, size in 115*115*70 mm (Magnetic flux density, 576mT; Juxing Magnetic Industry, China) mounted on a robotic arm (LBR Med 14 R820, KUKA, Germany) (Figure 1). The robot arm compensated for the weight of the heavy magnet and allowed effortless adjustment of the external magnet position, thus providing dynamic tissue countertraction based on the procedural requirement.

### 2.2 Study Design

This is an ex vivo, open label, randomized crossover study using porcine colon models. Endoscopists were invited to perform colonic ESD in two consecutive rounds. In each round, every endoscopist performed both magnetic countertraction (MAG‐ESD) and conventional ESD (C‐ESD) on paired specimens. The sequence of procedures within each round (MAG‐ESD first or C‐ESD first) was randomly assigned to minimize order effect. As this was an ex vivo study using animal specimens, formal ethical approval and prospective registration were not required per institutional policies. However, we have adhered to the CONSORT guidelines for crossover trails in reporting this study (Table S1).

### 2.3 ESD Procedure

**FIGURE 2:**
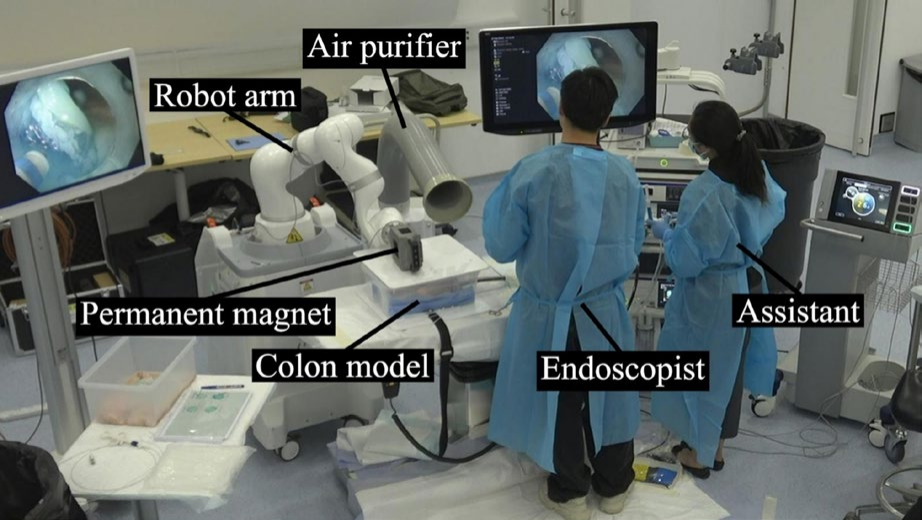
Setup of the magnetic countertraction system during ex vivo colon ESD model.

The procedures were performed using an ex vivo porcine colon model as described previously [10]. In brief, an explanted colon model ~30 cm in length was connected to an air‐sealed overtube (Guardus Overtube, BX00711147, Steris Healthcare, Ireland). The model was placed within a plastic box with a cover lid placed 15 cm above the colon. The purpose of keeping a 15 cm distance was to simulate the anticipated distance between the external magnet and the colon, taking into account an average abdominal wall thickness of 4.5 cm with an obese adult and the potential distance of the colon from the abdominal wall [11] (Figure 2). Thirty‐millimeter‐sized target lesions were marked at gravity‐dependent locations. ESD were performed with standard gastroscope (GIF‐EZ1500, Olympus Medical Corporations, Tokyo, Japan) and transparent straight hood, using needle type knife (Dual knife J, KD‐655U, Olympus Medical Corporations, Tokyo, Japan) and normal saline plus Indigo‐carmine as the standard lifting solution.

### 2.4 MAG‐ESD Procedure

**FIGURE 3:**
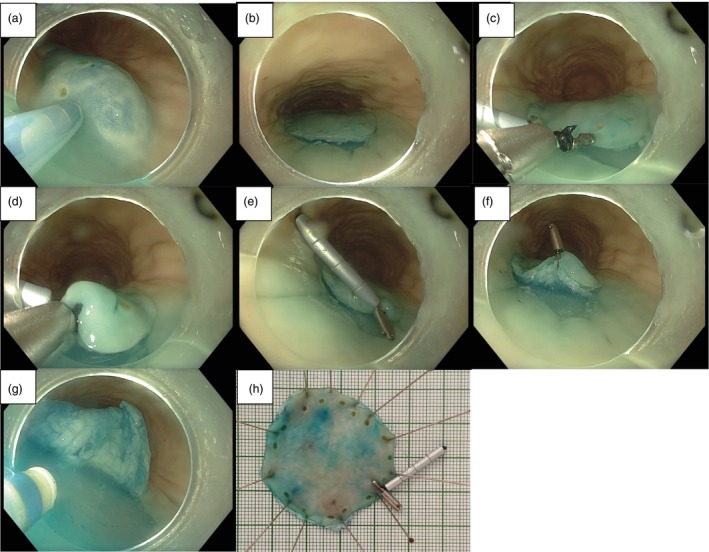
Procedure of MAG‐ESD with novel magnetic countertraction system. After the endoscope reached the pre‐marked lesion, (a) submucosal injection, (b) mucosal incision and trimming. (c–e) Internal magnetic retractor application on the near side of the lesion edge. (f) Establishment of magnetic countertraction. (g) Effect of magnetic countertraction system during the procedure. (h) The resected lesion with markings, pinned on a board.

After submucosal injection, a circumferential mucosal incision was performed around the marked lesion. The internal magnet retractor was then delivered through the endoscopic channel and the clip was deployed at the edge of the dissected lesion over the near side. The robotic arm mounted EPM was then moved by the endoscopist to the proximity of the target lesion and positioned to allow optimal internal countertraction by the retractor. The EPM was fully controlled and manipulated via the robotic arm during all procedures. Submucosal dissection was then continued and traction direction could be adjusted according to the intra‐procedural requirement. Upon completion of the procedure, the EPM would be moved away from the target area to disengage force to the internal magnet retractor, which would then be retrieved together with the specimen (Figure 3 and Video S1).

### 2.5 Conventional ESD

ESD would be performed using conventional techniques on the same model. During the procedure, no external countertraction method was allowed. Pocket creation, tunneling method, and underwater dissection were permitted at the discretion of endoscopists.

### 2.6 Outcomes

The primary outcome of the study was procedure time, defined from submucosal injection until the completion resection of the lesion. Secondary outcomes include en bloc resection, partial thickness muscle injury and full thickness perforation, defined as muscular layer damage and full thickness perforation of the muscle wall respectively, assessed by an independent assessor. In addition, endoscopists' workload measured by the NASA Task Load Index (NASA TLX) [12] was also collected immediately after each procedure. The NASA TLX is a multidimensional assessment tool that rates perceived workload to assess performance of a task. It consists of six sub‐scores of mental demand, physical demand, temporal demand, effort, performance and frustration, each allocated with a score of 0–100 on a visual analog scale, with a final total weighted score of 0–100 after weighting the relevant importance of each component [12]. A lower NASA TLX score indicates lower workload of the endoscopist.

### 2.7 Sample Size Estimation

Based on our pilot preclinical study, the procedural time for MAG‐ESD and C‐ESD was 16.2 ± 16.15 and 26.3 ± 9.54 min, respectively [9]. The initial sample size calculation, based on independent sample assumptions (Two‐sided alpha = 0.05 and power = 0.8), estimated that 72 procedures (36 per group) would be required. After consultation with a biostatistician, the sample size was revised to reflect the paired design. Using a paired *t*‐test (two‐sided alpha = 0.05, power = 0.8), a mean difference of 10.1 min and a standard deviation (SD) of the paired differences of 18.76 yielded a required sample size of 30 pairs, assuming a conservative within‐pair correlation of 0. With a sample size of 36 completed pairs, the study can accommodate a design effect. This sample size remains sufficient under reasonable intraclass correlation (ICC) values (e.g., ICC ≤ 0.2: design effect ≤ 1.2; ICC = 0.3: design effect = 1.63). Therefore, the achieved sample size provides adequate statistical power for detecting the anticipated effects.

### 2.8 Randomization

Immediately before each round of procedures, each endoscopist was randomized to start with either MAG‐ESD or C‐ESD using an online application independently. The concealed randomization sequence was generated online and allocated to each endoscopist by one of the study investigators (WSC). The purpose of this simple randomization was to minimize the effect of the learning curve from performing repeated procedures.

### 2.9 Statistical Analysis

All analytical procedures were determined post hoc based on the study design and data characteristics. Data were analyzed using SAS version 9.4 (SAS Institute Inc., Cary, NC). For the primary outcome of procedure time, linear mixed models with natural log transformation are applied to address right‐skewness. The model included group and sequence as fixed effects, with endoscopist included as a random intercept. Group‐by‐sequence and group‐by‐round interactions were both initially tested at α = 0.10 and removed from the final model due to insignificance (*p* = 0.71 and *p* = 0.55, respectively). Results were back‐transformed to the original scale; treatment effects are presented as geometric mean ratios (GMR) and geometric mean differences (GMD), with 95% confidence intervals (CI) calculated using delta methods. For the binary outcome of intra‐procedural adverse events including muscle injury and perforation, a generalized estimating equations (GEE) model with logit link and exchangeable correlation structure was used to account for within‐endoscopist clustering. Results are reported as odds ratios with 95% CI. Statistical significance was defined as two‐sided *p* < 0.05 for all analyses. Sensitivity analyses were conducted by including round sequence within each endoscopist as an additional fixed factor for all models. Subgroup analyses were performed by dividing the endoscopists into novice (< 80 experience in colorectal ESD) and expert (> 80 experience in colorectal ESD) based on a previous study [13].

## 3 Results

From August 2024 to June 2025, 72 ESD procedures (36 MAG‐ESD and 36 C‐ESD) were performed by 18 endoscopists in two rounds. Based on simple randomization, 20 pairs were randomized to start with MAG‐ESD and 16 pairs to start with C‐ESD. This slight imbalance was expected from unrestricted randomization and was not predetermined. All ESD procedures were successfully completed by the endoscopists, with an en bloc resection rate of 100%.

### 3.1 Primary Outcome Analysis—Procedure Time

Linear mixed model analysis revealed a significant main effect of group on procedure time (*p* = 0.0002). Sequence effect was not significant (*p* = 0.8168). The median procedure time was 62.2 min (range: 18.5–146.7) for C‐ESD and 45.8 min (range: 18–115.2) for MAG‐ESD. MAG‐ESD demonstrated a significant reduction in procedure time compared to group C‐ESD, with a geometric mean ratio of 0.796 (95% CI: 0.706–0.886), corresponding to a 20.4% reduction in procedure time (Table 1).

**TABLE 1:** Procedure outcomes of MAG‐ESD and C‐ESD.

Outcome measurement	MAG‐ESD (*n* = 36)	C‐ESD (*n* = 36)	Difference (95% CI)	*p*
Procedure time (min), median (range)	45.8 (18–115.2)	62.2 (18.5–146.7)	GMR: 0.796 (0.706, 0.886)	0.0002
Conventional technique, *n* (%)	—		—	—
Conventional		12 (33.3)		
Pocket creation method (PCM)		13 (36.1)		
Tunneling technique		2 (5.6)		
Underwater (Saline immersion) technique		9 (25.0)		
Magnetic retraction establishment time (min), median (range)	2.1 (0.8–11.6)	—	—	—
Complete en bloc resection, *n* (%)	36 (100)	36 (100)	—	—
Magnetic device failure, *n* (%)	0 (0)	—	—	—
Intra‐procedure complication, *n* (%)	5 (13.9)	14 (38.9)	OR: 0.782 (0.644, 0.949)	0.0127
Partial thickness muscle injury, *n* (%)	5 (13.9)	13 (36.1)	OR: 0.803 (0.673, 0.959)	0.0154
Full thickness perforation, *n* (%)	0 (0)	6 (16.7)	OR: 0.846 (0.726, 0.987)	0.0339
Weighted NASA TLX (Mean ± SD)	32.98 ± 12.77	52.79 ± 17.61	−19.81 (−25.42, −14.19)	< 0.0001
Mental demand	41.11 ± 18.09	63.19 ± 18.29	−22.08 (−28.19, −15.97)	< 0.0001
Physical demand	38.33 ± 17.40	56.53 ± 19.85	−18.19 (−24.63, −11.76)	< 0.0001
Temporal demand	36.81 ± 17.08	56.39 ± 19.88	−19.58 (−25.87, −13.29)	< 0.0001
Effort	39.03 ± 17.35	60.97 ± 21.27	−21.94 (−28.03, −15.85)	< 0.0001
Performance^a^	27.92 ± 10.72	45.00 ± 19.71	−17.08 (−23.28, −10.88)	< 0.0001
Frustration	29.17 ± 15.79	50.28 ± 22.99	−21.11 (−29.00, −13.22)	0.0244

*Note:* All comparisons use C‐ESD as reference group. All statistical comparisons were adjusted for sequence effect and accounted for within‐surgeon clustering using linear mixed models for continuous outcomes and generalized estimating equations for binary outcome.

Abbreviations: CI = confidence interval; GMR = geometric mean ratio; OR = odds ratio.

^a^ Lower score of performance indicates better performance rated by endoscopist.

### 3.2 Secondary Outcomes Analyses

GEE analysis revealed a significant effect of group on intra‐procedural adverse events (*p* = 0.0127). MAG‐ESD was associated with significantly lower odds of adverse events compared to C‐ESD (OR = 0.782, 95% CI: 0.644–0.949). Similar protective effects were observed for partial thickness muscle injury (OR = 0.803, 95% CI: 0.673–0.959) and full thickness perforation (OR = 0.846, 95% CI: 0.726–0.987). Notably, no full thickness perforation occurred in the MAG‐ESD group, compared to six cases (16.7%) in the C‐ESD group (Table 1).

Linear mixed model analysis showed a significant main effect of group on weighted NASA‐TLX score (*p* < 0.0001). MAG‐ESD demonstrated substantially lower weighted NASA‐TLX scores compared to C‐ESD, with a mean difference of −19.81 points (95% CI: −25.42 to −14.19). All NASA‐TLX subscales consistently showed significantly lower scores in the MAG‐ESD group (all *p* < 0.0001), with the largest reductions observed in mental demand (−22.08 points), effort (−21.94 points), performance (−17.08 points), and frustration (−21.11 points) (Table 1).

Sensitivity analyses including procedural round sequence as a fixed factor across all models showed that the primary findings were not altered, with all effect estimates remaining stable and statistical significance unchanged. Subgroup analysis (Table S2) revealed that the benefits of MAG‐ESD were predominantly observed among novice endoscopists, with significant reductions in procedure time (GMR 0.75, 95% CI: 0.64–0.86, *p* < 0.01), intra‐procedure adverse event (OR 0.68, 95% CI: 0.55–0.85), indicating substantially greater MAG‐ESD benefits for novices in procedural safety measures. Among experts, MAG‐ESD significantly reduced workload scores across all NASA TLX domains (all *p* < 0.01) but showed no significant advantage in procedure time or adverse events.

## 4 Discussion

Achieving adequate exposure of the submucosal dissection plane is essential for successful performance of ESD. Various countertraction methods have been developed, most involving clip assisted techniques to achieve a static countertraction force toward the opposite wall of the gastrointestinal lumen or the natural orifice [2, 3, 14]. Other reported methods include pocket creation method [15], tunneling technique, and underwater (saline immersion) dissection [16]. Our preclinical randomized trial, utilizing a novel magnetic guided countertraction system, demonstrated enhanced performance of colonic ESD, with reduced procedural time, complications, and endoscopists' workload.

Clip line‐assisted traction is one of the simplest countertraction methods. Yamasaki et al. [17] demonstrated in a small randomized trial shorter procedural time with line assisted traction, but its application in colorectal ESD is limited due to the need for insertion of pre‐formed loop through the endoscopic channel and the unpredictable direction of traction due to long distance from the anus. Clip‐band technique is another clip‐assisted technique commonly utilized, with commercially available devices such as S‐O clip. A single center randomized study showed potential benefit of S‐O clip in colorectal ESD with shorter procedural time [18]. However, a recent multicenter randomized study on traction‐assisted ESD using either clip line or S‐O clip method failed to show superiority over conventional method in procedural time and adverse event [19]. The main drawback of these methods was the lack of dynamic traction, as the traction direction could not be altered after application. The traction force of clip‐band technique may also diminish over time depending on the elasticity of the band and the distance to the opposite wall clip. The colonic lumen would need to be fully distended to allow maximum traction, potentially leading to patient discomfort. In contrast, magnetic‐guided countertraction systems provide dynamic traction based on the direction of magnetic field by the external magnetic device. Moreover, magnetic traction is not affected by gastrointestinal lumen distension and provides adequate traction even underwater.

Kobayashi and Gotoda first reported magnetic retraction device during gastric ESD. It consisted of an internal magnet anchor (1.0 × 1.0 × 1.5 cm) and an external electromagnetic control system [4, 5]. This system was used in a prospective pilot clinical trial of 25 gastric ESD, demonstrating 100% en bloc resection without adverse event. The main limitation was the cumbersome setup of the electromagnetic control system. Matsuzaki et al. [6] reported another system using internal neodymium magnet anchor and an external permanent magnetic control device, achieving en bloc resection in all 50 patients undergoing gastric ESD. While this novel system overcame the complexity of setup, the size of the internal magnet prevented introduction through the endoscope channel, limiting its applicable in colorectal ESD [20]. In contrast, our system is more advantageous especially for colorectal ESD by allowing introduction of internal magnetic anchor through the endoscope channel, without need for scope withdrawal. The robotic arm mounted external magnet also enabled effortless adjustment of countertraction direction through free movement of the magnet while supporting magnet weight. Due its simplicity, our traction device only took 2 min for establishment, compared to a median of 8 min in previous study [20]. A case report of using stainless steel internal anchor was described by Matzusaki et al. [8] in a successful colorectal ESD which could be introduced through the channel of the endoscope. The use of stainless steel anchor could be an alternative, but the magnetic retraction force could be significantly lower and thus providing inadequate traction in other scenarios. The lack of polarity with the stainless steel would also limit freedom of movement compared to that provided by an internal magnetic anchor. In our ex vivo experiments, no interference between the internal magnet and the endoscope tip was observed.

Through standardizing the setting of ESD in a porcine colon model, we demonstrated unequivocal results that magnetic countertraction assisted ESD could achieve better procedural efficiency and safety, especially among novice endoscopists. The difference among experts was less significant, most likely as they have developed additional techniques to overcome difficulties encountered in a conventional method without countertraction. We also did not directly compare other countertraction methods such as SO‐clip with our novel traction method. A prospective clinical trial is now ongoing to evaluate the efficacy and safety of our novel magnetic countertraction system during gastric and colorectal ESD (Clinicaltrial.gov, NCT06962293). Moreover, the cost‐effectiveness of the novel magnetic countertraction system should also be evaluated.

Our study has several limitations. First, the ex vivo model could not completely resemble the clinical situation because there was no blood perfusion or active bleeding. Since hemostatic interventions were unnecessary, the dissection speed was faster than that reported in clinical studies. Secondly, the perforation rate was higher than that reported in clinical studies, likely due to a larger proportion of novice endoscopists performing ESD in a simulated environment. Thirdly, although the control arm consisted of the pocket creation method and underwater procedure, we could not include other clip‐related traction techniques due to limited laboratory resources; direct comparison of MAG‐ESD versus other traction methods could not be achieved. Finally, complete blinding is impossible, as traction clips were visibly present during MAG‐ESD procedures. For both the endoscopist and independent assessor, this inherent visibility may have introduced observer bias in outcome assessment, including a risk of favoring MAG‐ESD on the procedural outcomes and workload assessment scores.

In conclusion, we developed a novel robotic magnetic countertraction system for colorectal ESD and demonstrated shorter procedural time, lower complication rates, and endoscopist‐reported workload in an ex vivo randomized controlled trial. Further prospective clinical study is ongoing to evaluate the performance of the system in real‐world clinical situations.

## Author Contributions

**Hon Chi Yip, Wai Shing Chan**, and **Zheng Li:** study concept and design. All investigators: data acquisition and administrative support. **Hon Chi Yip, Wai Shing Chan, Man Yee Yung, Zhaoyi Zhu, Yee Kit Tse**, and **Zheng Li:** data analysis and interpretation. **Wai Shing Chan, Siew Fung Hau**, and **Hon Chi Yip:** manuscript drafting. **Zhaoyi Zhu, Yee Kit Tse, Philip Wai‐yan Chiu**, and **Simon Siu‐man Ng:** critical revisions. **Hon Chi Yip:** guarantors of the article.

## Funding

This research is supported in part by the Hong Kong Research Grants Council with project No. C4042‐23GF, 14214322, and 14200623, and in part by the Multi‐scale Medical Robotics Center, AIR@InnoHK MRC, Innovation and Technology Commission (ITC).

## Conflicts of Interest

H.C.Y. served as lecture speaker for Olympus Medical System Co Ltd., Cornerstone Robotics Ltd., Medtronics Hong Kong Medical Ltd., Creo Medical Co. Ltd. L.H.‐S.L. has research collaborations with Olympus Co. Ltd., ASUSTeK Computer Inc. and GenieBiome Ltd., and served as advisory board for AstraZeneca and GenieBiome Ltd., and served as lecture speaker for Olympus Co. Ltd., Boston Scientific Co. Ltd., Pfizer Inc., and GenieBiome Ltd. P.W.y‐C. has research collaboration with Olympus Co. Ltd. and Boston Scientific, and served as an advisor for EndoVision and EndoMaster, and served as a lecture speaker for Olympus Medical System Co. Ltd.

## Supporting information


**Table S1:** CONSORT checklist for crossover trials.

**Table S2:** Subgroup analysis of novice and expert endoscopists.

**Video S1:** Short video of MAG‐ESD procedure.

## Data Availability

Complete anonymized data, analysis methods, and research materials will be made available to other researchers at the time of publication for 5 years according to specific request.
