# Supplementary material for: Novel Magnetic Countertraction Enhanced Performance in Colonic Endoscopic Submucosal Dissection: An Ex Vivo Crossover Study (With Video)
Source: Dig Endosc. 2026 Feb 10;38(2):e70120. doi: 10.1111/den.70120 (PMC12887612; doi:10.1111/den.70120)
Supplement: Supplementary file 2 — Table S2: Subgroup analysis of novice and expert endoscopists. [file DEN-38-0-s001.docx]

**Supplementary Table 2:** Subgroup analysis of novice and expert endoscopists

| MAG-ESD Treatment effect | Novice  (n=52) | Expert  (n=20) | p-value for heterogeneity |
| --- | --- | --- | --- |
| Procedure time, GMR (95%CI) | 0.75 (0.64 – 0.86)^**^ | 0.93 (0.80, 1.05) | 0.1075 |
| Dissection speed, GMR (95%CI) | - | - | - |
| Intra-procedure complication, OR (95%CI) | 0.68 (0.55 – 0.85)^**^ | 1.11 (0.93, 1.32) | 0.0008 |
| Partial thickness muscle injury, OR (95%CI) | 0.71 (0.58,0.87)^**^ | 1.11 (0.93, 1.32) | 0.0011 |
| Full thickness perforation, OR (95%CI) | 0.79 (0.65 – 0.97)^*^ | NC^†^ | 0.0257 |
| Weighted NASA-TLX, mean difference (95%CI) | -16.63 (-26.51, -6.76)** | -21.03 (-28.04, -14.01)** | 0.4861 |
| Mental demand, mean difference (95%CI) | -19.50 (-32.79, -6.21)** | -23.08 (-30.30, -15.85) ** | 0.6029 |
| Physical demand, mean difference (95%CI) | -23.00 (-34.67, -11.33)** | -16.35 (-24.12, -8.58)** | 0.3562 |
| Temporal demand, mean difference (95%CI) | -23.00 (-36.63, -9.37)** | -18.27 (-25.65, -10.89)** | 0.5030 |
| Effort, mean difference (95%CI) | -18.00 (-29.87, -6.13)** | -23.46 (-30.93, -15.99)** | 0.4242 |
| Performance, mean difference (95%CI) | -10.00 (-17.83, -2.17)** | -19.81 (-27.81, -11.80) ** | 0.1550 |
| Frustration, mean difference (95%CI) | -15.50 (-27.42, -3.58)** | -23.27 (-33.31, -13.23) ** | 0.3787 |

^*^P < 0.05, ^**^P < 0.01 for within-subgroup treatment effects.

^†^NC = Not calculable (no events in either group).

**Note:** CI = Confidence Interval; OR = Odds Ratio; GMR = Geometric Mean Ratio. All within-subgroup comparison used C-ESD as reference group. All statistical comparisons were adjusted for sequence effect and accounted for within-surgeon clustering using linear mixed models for continuous outcomes and generalized estimating equations for binary outcomes.
